# Food Allergy a Constant Concern to the Medical World and Healthcare Providers: Practical Aspects

**DOI:** 10.3390/life11111204

**Published:** 2021-11-08

**Authors:** Lucia M. Sur, Ionel Armat, Emanuela Duca, Genel Sur, Iulia Lupan, Daniel Sur, Gabriel Samasca, Cecilia Lazea, Calin Lazar

**Affiliations:** 1 Department of Pediatric I, Iuliu Hatieganu University of Medicine and Pharmacy, 400006 Cluj-Napoca, Romania; Sur.Maria@umfccluj.ro (L.M.S.); cecilialazea@umfcluj.ro (C.L.); calinlazar@umfcluj.ro (C.L.); 2 Children Emergency Clinical Hospital, 400370 Cluj-Napoca, Romania; armat_95ionel@yahoo.com (I.A.); Floca.Emanuela@umfcluj.ro (E.D.); surgenel@yahoo.com (G.S.); 3 Molecular Biology Department, Babes Bolyai University, 400084 Cluj-Napoca, Romania; iulia.lupan@ubbcluj.ro; 4 The Oncology Institute “Prof. Dr. Ion Chiricuta”, 400015 Cluj-Napoca, Romania; Daniel.Sur@umfcluj.ro; 5 Department of Allergology and Immunology, Iuliu Hatieganu University of Medicine and Pharmacy, 400006 Cluj-Napoca, Romania

**Keywords:** immunopathology, perspective, management, children, adult, diagnosis

## Abstract

Food allergy (FA) is a condition with a growing incidence and is a constant concern for the medical world and healthcare providers. With potential symptoms including anaphylaxis, in the event of an allergic reaction the patient’s life may well be endangered. The diagnosis of FA is a continuous challenge because mild cases tend to be ignored or diagnosed late and young children with allergies are cared for by parents, who are not always able to accurately interpret symptoms. It is very important to be able to differentiate FAs from food intolerance and toxic reactions to food. An accurate diagnosis is required to provide personalized management of an FA. More sophisticated and accurate diagnostic tests, including component diagnosis and epitope reactivity, allow the provision of a directed diagnosis, a more accurate therapeutic approach, and a useful prognostic evaluation. Tests used in current practice include the specific search for serum IgE, elimination diets, oral food challenges, single, blind, and double-blind (DBPCFC) tests, as well as skin tests. The risk of anaphylaxis can be assessed by molecular diagnostics/component-resolved diagnosis (CRD) and by conducting a basophilic activation test (BAT). These tests allow a planned, personalized treatment based on molecular and clinical profiles. CRD can determine the individual profile of allergic molecular reactivity and enable the formulation of a prognostic judgment. Our article highlights the importance of knowing the immune mechanisms, diagnostics, and immunotherapies in FAs. Starting from observing exposure to food allergens, to identifying allergic reactions, analysing the severity of clinical manifestations, noting the possibilities of diagnosis, and illustrating adequate management strategies.

## 1. Introduction

Although the vast majority of people can consume food without clinical manifestations, certain foods can cause reactions in people who are sensitive to one or another of their ingredients. These food reactions can lead to anaphylactic shock and even death if proper measures are not taken. To establish the diagnosis of a food allergy (FA), a combination of methods is needed, such as a detailed medical history, clinical symptoms, laboratory tests, and in not a few cases an oral food challenge (OFC). It is known that the elimination of the allergen is very important in the management of food allergies, but without sufficient consideration of the evidence needed to build an accurate allergy profile, inappropriate elimination diets may be introduced which could lead to nutritional deficiencies. For this reason, the diagnosis of an FA should include all available methods and techniques that allow for a correct FA assessment [1,2,3,4,5].

Probably due to processed foods, industrial preparation procedures, pollution, and the introduction of food additives, FAs have seen a steady increase, particularly in developed countries [6,7,8,9,10,11,12,13]. As risk factors for food allergies, we also mention genetic predisposition, which is proven by the fact that FA is more common in people with atopy. 

This increase has also been observed in developing countries that have imported many food preparation methods [14].

### 1.1. Food Allergy Prevalence

The prevalence of food allergies is difficult to assess because it varies with age, environment, and eating habits [15]. The prevalence of FAs is higher in infants and young children 6–10% [4,16,17,18]. Studies conducted in Europe have found a prevalence of food allergies to common foods between 5.6% and 6.4% [19]. A review of fish allergies showed a prevalence of 7% [19,20]. Regarding egg allergies, a cohort study from nine countries reported a prevalence in the range of 1.23–1.5% [21], the highest incidence being reported in the United Kingdom (2.1%), the lowest in Greece (0.07%) [19,21]. Various studies have shown that approximately 2.5–3% of infants are allergic to cow’s milk [16,17,18]. In some children, these food allergies may persist for the rest of their lives while others may gain tolerance along the way (Table 1).

It has also been found in developed countries that the frequency of allergies has generally increased so that one in four Europeans suffers from some form of allergy, and FA has been described by some authors as the second wave of the “allergic epidemic” [22,23].

### 1.2. Food Allergens

**Figure 1:**
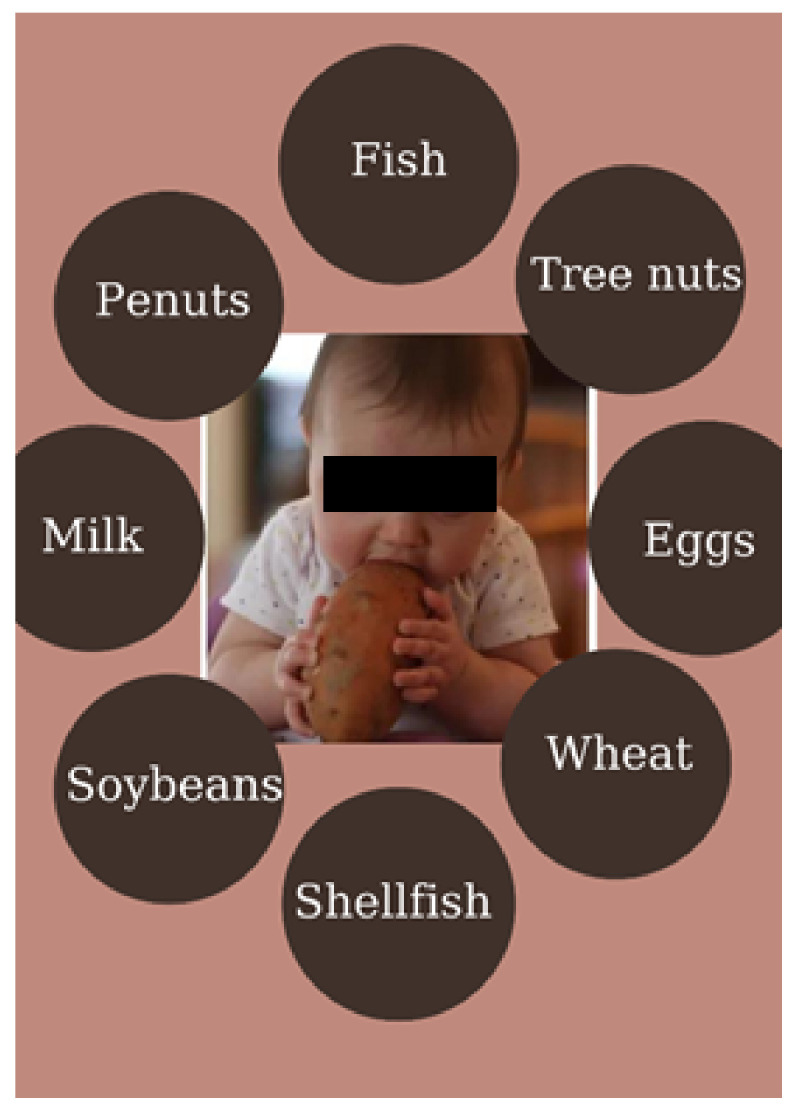
“Big eight” food allergens.

There are eight major food allergens referred to in the literature as the “big eight” (Figure 1) which are responsible for up to 90% of food allergies: milk, eggs, nuts, peanuts, fish, shellfish, wheat, and soy [19,20,21]. FAs are more common in children than in adults. Peanuts and tree nuts trigger up to 87% of deaths; other foods, including milk and seafood, cause mortality in some countries [22].

The phenomenon of cross-reactivity between different food species has also contributed to the increased prevalence of food allergies [24,25]. Molecular biology techniques have enabled the discovery of a variety of cross-reacting allergens among foods, such as tropomyosins in crustaceans and arachnids, lipid transfer protein in peaches, apples, peanuts and tomatoes; parvalbumins in fish; class I chitinases in latex and wheat; bovine IgG in milk and lamb; and profilin in plums, birch pollen, and latex [26].

## 2. Physiopathology

Our immune system plays an essential role in maintaining tolerance to harmless food antigens. IgE-mediated FAs occur secondary to the loss of the integrity of the immune elements responsible for differentiating benign food allergens from pathogens [15,27,28]. If these food antigens cross the mucosal barrier they are taken up by dendritic cells and the production of suppressive cytokines, such as IL10, is triggered. After that, the naive T transformation into regulatory T cells occurs with the suppression of Th2 and the increase of Th1, leading to the production of IgA and IgG 4 [5,11,15]. Sensitization to food allergens translates into the appearance of IgE specific to detectable foods that ultimately lead to the development of clinical FAs. When food crosses the disturbed digestive barrier, proinflammatory cytokines, such as IL25 and IL33, are released, which activate dendritic and lymphoid cells [1,15,27]. These activated cells lead to naive T transformation with the acquisition of the Th2 cellular phenotype, which stimulates B cells to produce food allergen-specific IgE [28,29,30]. This sensitization is the recognition of a food antigen as a threat. There are several key components of the immune system involved in the development of tolerance and awareness. These components are the epithelium, innate immune cells, T cells, B cells, and the cells that carry out the allergic response (mast cells, eosinophils, and basophils) [15,28]. It is important to keep an epithelial barrier intact to prevent allergen sensitization and manifestations of FAs [1,15].

Differentiated Th2 can secrete proinflammatory cytokines, such as IL5 and IL13, and promote cell differentiation, affecting eosinophils and basophils [14,15,16]. Mast cells and basophils participate in the allergic process. In the presence of IL10 and TGF β, IgA can be produced through the activity of B cells that maintain tolerance [1,11], the mechanism is not fully known. IL4 produced by TH2 induces the production of antigen-specific IgE in B cells. Tregs cells (Tr1 cells) mediate antigen-specific T cell tolerance. The main cytokine produced by Tregs is IL-10, which is responsible for suppressing the effector T cell response. IL-10 has an immunosuppressive capacity very important for the establishment of peripheral tolerance to allergens. Exposure to Ag occurs by attaching IgE to the surface of mast cells and basophils with the release of preformed mediators in circulation. Mediators that are involved in anaphylaxis are histamine, tryptase, platelet-activating factor, prostaglandins, and leukotrienes [9,15].

The pathophysiology of non-IgE-mediated FAs is less understood. An association between IgE-mediated and non-mediated allergy has been described [1,15].

Alpha-gal syndrome (AGS) is an immune-mediated disorder caused by hypersensitivity responses to glycan galactose-alpha-1,3-galactose (alpha-gal). A significant difference between usual food allergies and AGS is the reaction time, with symptom onset occurring two or more hours after alpha-gal consumption [31].

## 3. Classification of Food Allergic Reactions

The European Academy of Allergy and Clinical Immunology (EAACI) has developed a guide to food allergies (Table 2). According to the guide’s proposals, allergies are classified into IgE-mediated, mixed IgE, and non-IgE mechanisms [15,32].

Another recent classification of the FA is that presented in the Japanese Pediatric Guideline for Food Allergy (JPGFA), which classifies food allergies into four major categories [1].

### 3.1. Neonatal and Infantile Gastrointestinal Allergy

This type of allergy manifests in the neonatal period, the most frequently incriminated food being cow’s milk. Specific antigen lymphocyte stimulation tests are positive in most cases, which demonstrate the cellular, non-IgE mechanism behind this type of allergy. The diagnosis is supported by the appearance of digestive manifestations after ingestion of the causal food, the disappearance of the symptoms after the removal of the incriminated food, and a positive test of food challenge.

### 3.2. Infantile Atopic Dermatitis Associated with and Aggravated by Food Allergies

This is the most common FA in childhood and the most commonly incriminated foods are eggs, cow’s milk, soy, and wheat. The pathogenic mechanism is IgE-mediated.

### 3.3. FA with Immediate Manifestation

The onset period for this FA can be noticed during childhood, adolescence, and even adulthood. The most common foods with immediate allergic potential, especially in infants are eggs, cow’s milk, peanuts, fish, and shellfish. 

### 3.4. Special Type

These are in turn divided into two subtypes, both being IgE-mediated:

Food-dependent exercise-induced anaphylaxis (FDEIA). This occurs during exercise, after ingesting allergenic food. It is often caused by ingesting wheat or shellfish products. It is more common in adolescence. It is triggered two hours after food intake, during sustained exercise;

Oral allergic syndrome (OAS): It occurs after ingestion of fresh fruits and vegetables, the food losing its allergenic character after thermal preparation. It is manifested by paresthesia in the oral cavity, pruritus, and swelling of the lips and tongue after eating raw foods. It occurs more frequently in people with atopy, and with pollen especially [32]. In infants and young children, FA can cause weight loss, neuro-mental disorders, agitation, lack of concentration, headaches, and migraines. There are many gaps in our understanding of the pathophysiology of non-IgE-mediated FAs. Eosinophilic esophagitis (EoE) and atopic eczema are associated with mixed, IgE, and non-IgE-mediated allergies. Associations between atopic eczema and the presence of gastrointestinal symptoms have been highlighted by Latcham and colleagues, which results were later confirmed by Meer and collaborators, according to whom 40% of children with gastrointestinal allergies induced by IgE-mediated proteins also had atopic eczema [33,34]. The same group of researchers showed that there may be atopic associations in IgE- and non-IgE-mediated allergies, and mentioned asthma and rhinitis specifically. These findings suggest that non-IgE-mediated allergies are multisystemic disorders. Recently, many other non-IgE-mediated entities have been analyzed, such as food protein-induced enterocolitis syndrome (FPIES), food protein-induced allergic proctocolitis (FPIAP), and food protein-induced proctocolitis (FPE). Pathologies with overlapping symptoms include EoE, eosinophilic gastroenteritis, and eosinophilic gastroenterocolitis. Although the phenotype is atopic, the underlying mechanism in these diseases remains to be investigated. Eosinophilic gastrointestinal disorders are primarily polygenic allergic disorders involving the type II mechanism and Th lymphocytes. Eosinophils produce strong pro-inflammatory effects by releasing lipid mediators and cytokines. Elevated levels of eosinophils occur in the lamina propria, these eosinophils being under the influence of IL-5 accumulated in the esophagus. In this type of reaction, dietary exclusions are essential but not sufficient for the control of symptoms, requiring pharmacological adjustment [1,11,35]. In the syndrome of enterocolitis induced by food proteins, the infant presents abundant vomiting or diarrhea, pallor, and lethargy, which appear from two to four hours after food ingestion. Usually, this entity is misdiagnosed but the evolution is good, and the symptoms can disappear until the age of three years. In addition to the digestive symptoms that appear in these children, eating difficulties can also occur as well as extraintestinal manifestations suggesting that this disorder is a systemic one. FAs can also play an important role in other gastrointestinal disorders, such as persistent colic and gastroesophageal reflux, especially if therapies prove ineffectual and patients suffer persistent constipation. These conditions can be caused by a milk allergy, which is not mediated by IgE. As these conditions are not associated with other IgE-mediated diseases, performing a specific serum IgE test will not support the diagnosis. Long term, these non-IgE-mediated conditions can cause functional intestinal problems and the initiation of allergic march [36,37]. In the allergic march the first manifestations are allergy to cow’s milk protein then atopic dermatitis (eczema), allergic rhinitis, and asthma. There are functional gastrointestinal disorders in children as well. Functional gastrointestinal disorders are caused by altered intestinal physiology in interaction with psychosocial factors. For a better understanding of this issue, the Rome III criteria were developed. These functional disorders are irritable bowel syndrome, functional bloating, constipation, and functional diarrhea.

## 4. Clinical Manifestations

Manifestations of FAs can occur at different levels: skin, eye, gastrointestinal, cardiovascular, respiratory, neurological, and systemic [2,38,39].

### 4.1. The Cutaneous Manifestations Translate into Urticaria, Pruritus, Rash, and Accentuation of the Symptoms of Atopic Dermatitis. Depending on the Severity of the Lesions, We Can Classify the Symptoms in Three Degrees of Severity

- Grade I: skin manifestations such as erythema, urticaria, and pruritus are localized.
- Grade II: generalized skin manifestations of moderate intensity.
- Grade III: generalized skin manifestations of high intensity.

### 4.2. Ocular Manifestations

- Conjunctival Erythema.
- Pruritus.
- Tear Hypersecretion.
- Periorbital Edema.

### 4.3. Gastrointestinal Manifestations Are Bloating, Abdominal Cramps, Nausea, Vomiting, and Diarrhea. Recent Studies Introduced Three Other Degrees of Severity

- Grade I: Feeling of discomfort in the oral cavity and throat, mild abdominal pain, nausea. These manifestations are localized and of low intensity.
- Grade II: Moderate abdominal pain, sore throat, and recurrent diarrhea with vomiting.
- Grade III: Severe abdominal pain, vomiting, and uncontrollable diarrhea.

### 4.4. Cardiovascular Manifestations: Hypotension, Palpitations, Tachyarrhythmias, or Rhythm Disorders. They Can Be Divided into Two Degrees of Severity

- Medium form: Hypotension tachycardia.
- Severe form: Dysrhythmia, hypotension, severe bradycardia.

### 4.5. Respiratory Manifestations: Rhinorrhea, Nasal Congestion, Sneezing, Wheezing, Dyspnea, Sometimes Even Glottic Edema, Cough. They Can Be Divided into Three Degrees of Severity

- Grade I: intermittent cough, rhinorrhea, nasal congestion and sneezing, lack of wheezing, and dyspnea.
- Grade II: Repetitive cough, accentuated wheezing.
- Grade III: Persistent cough, wheezing, and dyspnea with SaO2 below 92%.

### 4.6. Neurological Disorders: Headache, Drowsiness, Vertigo, Anxiety, Incontinence, Loss of Consciousness. These Were Divided According to Severity into Three Degrees

- Grade I: changes in daily behavior.
- Grade II: moderate and drowsy headache.
- Grade III: fatigue, anxiety, loss of consciousness.

### 4.7. Systemic Manifestation

- Anaphylactic Shock with a Feeling of Weakness.
- Headaches.
- Visual Disturbances.
- Ear Noises, Sometimes.
- Vascular Collapse.

## 5. Association with Other Diseases

There is a possibility of the progressive development of allergic diseases. The allergic march is the progressive chain of several allergic diseases, from allergy to milk proteins around the age of 2–3 months of life to atopic dermatitis, also present in infants, followed by rhinitis and asthma during childhood. In the new conception of allergic march, allergic gastrointestinal diseases are also included [40,41]. These have been associated with the ingestion of specific foods, especially cow’s milk [42]. Although these clinical associations were found, the immune mechanism could not be established. If these diseases are diagnosed, the association between them and the ingestion of a certain food should be analyzed and the involvement of an FA considered. Some of these gastrointestinal diseases have increased greatly in incidence; thus, EoE is more common in adults. They have been reported cases of EoE in pediatrics, however, symptoms vary according to the patient’s age. Among the symptoms, we mention eating disorders, vomiting in infants and schoolchildren, abdominal pain, and swallowing disorders in school-age children and adolescents.

## 6. Diagnosis of Food Allergies

### 6.1. Medical History 

The first step in diagnosing an FA is to obtain a detailed history of clinical manifestations. It must include a complete list of foods suspected of triggering symptoms, the minimum dose at which clinical manifestations occur and the manner of preparation (baked, fried, boiled, and raw). Another important aspect is the reproducibility of symptoms at repeated exposure. Medical history should include factors that potentiate the onset of symptoms, such as exercise, association with alcohol, or nonsteroidal anti-inflammatory drugs (NSAIDs). Family history should be determined.

### 6.2. Skin Prick Tests (SPT)

A skin prick test consists of the insertion/inoculation of a small amount of allergen into the surface layer of the skin in order to evaluate the body’s response to the allergen tested. Skin tests can be performed even in infants from the age of six months [43]. Medications such as antihistamines, antiallergics, or steroids should be discontinued at least three days before a prick test [1,6]. A positive result indicates the presence of specific IgE antibodies [1]. It has been observed that some patients with negative IgE antibodies in serum may exhibit positive Prick test phenomena; this is encountered especially in infants [5,6,44]. The main disadvantage of skin tests is their low specificity, resulting in a high percentage of false-positive results. In the case of food allergies, the sensitivity of the skin test is approximately 90%. Therefore, the best method to rule out the presence of FAs is a negative Prick test while a positive test is not enough for diagnosis [27].

### 6.3. Antigen-Specific Ige(S-Ige) Antibodies in the Blood

Elevated IgE is a marker of atopy. They occur through an imbalance of helper T lymphocytes by increasing Th2, which stimulates B lymphocytes with excessive production of IgE. The IgE value is increased in atopic patients [6]. The determination of specific IgE antibodies in the patient’s serum is one of the most widely used methods to document an FA [8]. The presence of elevated levels of IgE titer specific to a particular food suggests either direct sensitivity to the allergen or a cross-reaction [6,8,11]. The measurement of specific IgE is not a direct quantitative estimate of antibody molecules; the results used for diagnosis and monitoring are dependent on this serum concentration [33]. The interpretation of the results is made according to probability, sensitivity, and specificity curves [1,6].

### 6.4. Patch Testing

This test is used to diagnose non-immediate IgE-mediated allergies. Allergens contained in special strips that are applied to the skin produce specific manifestations (erythema, edema, and induration); results are assessed according to controls. In one study, the atopy patch test predicted the results of an OFC in 28 out of 33 cases. There was a 100% negative predictive value for the atopy patch test [45].

### 6.5. Basophil Activation Test

This is a test with high specificity and negative predictive value. This represents a quantitative estimate of IgE-dependent basophilic activation. In place of histamine release, the expression of the activation markers CD63 and CD203c are measured by flow-cytometry [1]. The main advantage of a basophil activation test over IgE-specific antibodies is its high specificity, which supports the diagnosis of FA when the test is positive [8]. For instance, basophil activation in a test for peanut allergies showed a specificity of 96% in a discovery cohort and 100% specificity in a validation cohort [43]. 

### 6.6. Elimination Diets

Elimination diets involves the elimination of foods that are suspected of being allergenic from the daily diet. A correct medical history, specific IgE antibodies, and an oral challenge test are needed to establish the allergy conclusively to avoid the adoption of an unbalanced diet through unjustified dietary restrictions which could lead to nutritional imbalances.

### 6.7. OFC Test

OFC tests currently represent the gold standard in the diagnosis of food allergies. These tests consist of consuming a small quantity of the incriminated food as an allergen and noting the appearance or non-appearance of the symptoms. The test is recommended to be performed under medical supervision as there is a risk of severe allergic reactions, including anaphylactic shock. The role of this test is primarily diagnostic. By identifying the causal allergen, the definite diagnosis of an FA and infantile atopic dermatitis associated with an FA can be established [43,44]. By identifying the threshold level at which the symptoms appear, the oral challenge test has an important role in the desensitization process by determining the safe dose of foods [43,45]. The test result may be influenced by certain doses of drugs, which is why it is necessary to discontinue treatment before investigating (Table 3).

In practice, the test is applied in three forms: open food challenge, single-blind food challenge, and double-blind placebo-controlled food challenge [34,35,46].

1. Open food challenge. This test involves ingesting the food in its natural form. It is used in cases where anamnesis and laboratory explorations suggest that there is a small probability that the incriminated food is the one responsible for the symptoms. However, supervision is recommended for 1–2 h after the test. Given that the patient expects an allergic reaction, the results may be influenced by them having this knowledge and thus exhibiting the symptoms unconsciously.
2. Single-blind placebo-controlled food challenge. The test provides more objective results compared to the previous one because the food is not served in its natural form, it is hidden in a capsule [46,47,48]. The control food (placebo) is chosen so that it is as similar as possible in taste, smell, and texture. In this way, the patient cannot distinguish whether or not the administered substance contains the allergenic food.
3. Double-blind placebo-controlled food challenge. This consists of the administration of the incriminated food and a placebo. The difference between a single-blind placebo-controlled food challenge and this test is that the examiner does not know the difference between placebo and allergen [46]. It is the gold standard in the diagnosis of FAs. The test is used more frequently in specialized studies and less often in current practice due to the complexity of its execution.

**Figure 2:**
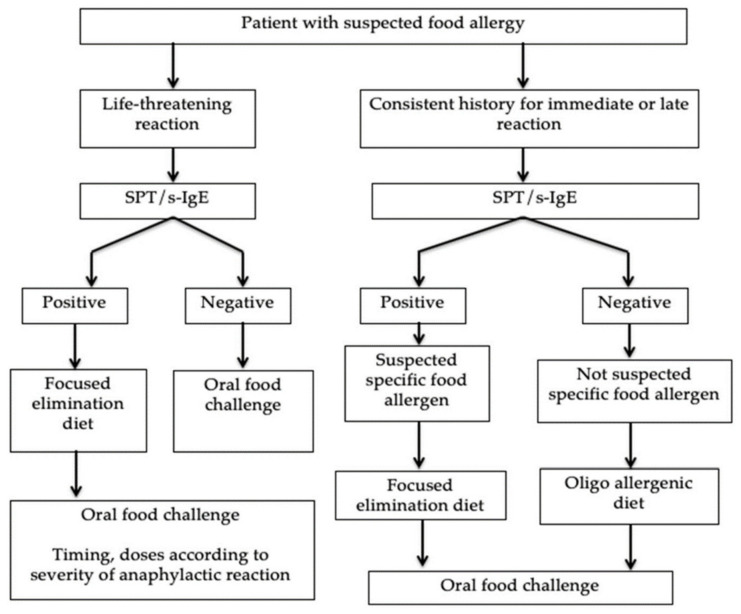
Diagnostic protocol for FAs.

The first line of diagnosis in the evaluation of a patient suspected of a food allergy is SPT and s-IgE. Depending on the result, the next step towards diagnosis is an OFC or an elimination diet for diagnostic purposes. The diagnostic protocol according to Muraro et al. is shown in Figure 2 [15,48].

## 7. Prevention and Treatment of FAs

### 7.1. Prevention

FA prevention is a very important goal in the management of these allergies. It can be achieved through strict and rigorous control of food, as well as by analyzing allergens by the ELISA technique or real-time PCR [36,37,49]. It is very important that all labels contain all the components of the food, as only trace amounts of certain foods can trigger allergic reactions in the sensitized. Another important aspect is the education of patients and their families, as well as food producers regarding the risks of using foods with high allergenic potentials. Regarding the exhibition of behaviors associated with a chronic disease, all the possible triggers of clinical manifestations must be determined and seen from an expansive point of view. The most useful preventive treatment is to eliminate the allergen from the diet and replace it with another food that is safer from an allergenic point of view. It is important to avoid food combinations with allergenic potential. We believe that adequate education along with knowledge about the disease in question and the allergenic foods associated with it can significantly reduce the frequency of emergencies caused by FAs.

### 7.2. Emergency Treatment

Depending on the severity of the clinical manifestations, emergency treatment will be customized according to the degrees of severity. Intramuscular adrenaline will be applied to all cases of third-degree severity. At the second degree of severity, adrenaline will be administered in the following situations: if there is a history of severe anaphylaxis, if the symptoms have shown progressive development, if circulatory signs appear, or if respiratory signs cannot be relieved with inhaled bronchodilators [1,29,50]. If a diagnosis of anaphylaxis is made, treatment must start immediately, and the vital signs will be followed. The dose of adrenaline typically administered is 0.01 mg/kg; the maximum dose for patients over 12 years is 0.5 mg and for patients under 12 years the maximum dose is 0.3 mg. If symptoms do not improve after multiple intramuscular administrations (up to three), the administrations will continue intravenously [51,52]. Body position is very important in anaphylaxis management. The patient will be made to lie in the lateral decubitus position with their head higher to avoid aspiration syndrome. The administration of oxygen is necessary and will be carried out with an O2 mask in the amount of 10 L/min. Intravenous rehydration and rebalancing will also be performed. As such, 10 mL/kg of Ringer’s solution or saline will be administered at 5–10 min intervals. In case of respiratory disorders, BLS procedures will be followed. They will monitor vital signs that will be reassessed frequently and regularly.

### 7.3. Immunotherapy

Immunotherapy with food allergens aims to induce a specific antigen immune tolerance in FA [4,53,54]. This immune tolerance translates into the patient’s ability to consume foods that in the past triggered allergic reactions without developing the characteristic symptoms. This is achieved by repeated administration of food allergens in progressively increasing doses [55,56]. Currently, three methods are used for administration:

#### 7.3.1. Oral Immunotherapy (OIT)

This therapeutic method involves the consumption of small amounts of the incriminated food [57,58]. The dose of food is gradually increased. At present, no exact protocols have been established regarding the doses of initiation, maintenance, and duration of treatment. The OIT is effective in inducing tolerance, but further studies are needed on the long-term effects. In terms of safety, most of the side effects reported have been mild; however, the risk of anaphylaxis is real [1,6,59]. To increase the safety profile of the OIT and at the same time increase its effectiveness, adjuvant treatment with Omalizumab is recommended. Another problem with OIT is the age at which treatment can begin. Given the fact that due to the risk of side effects this treatment can be a source of anxiety, it has been recommended that it is applied to children over six years old. Opinions are divided on the minimum age, however, so further studies and protocols are needed to address this issue.

#### 7.3.2. Sublingual Immunotherapy (SLIT)

SLIT consists of the sublingual administration of liquid preparations containing proteins from food that is allergenic. It is less effective than the OIT, especially in achieving a long-lasting tolerance because the maximum amount of allergen that can be administered by this method is a few milligrams [60,61]. In terms of its safety profile, this therapy was found to be superior to the OIT. SLIT is recommended for patients who do not tolerate OIT or as a method of initiating desensitization. After a period of sublingual administration, a switch is made to oral administration of food, thus mitigating the risk of severe side effects within the OIT. As a perspective, the development of quantified food extract-isolated proteins may be the solution to overcome the limitation of the method in terms of the maximum amount of protein that can be administered.

#### 7.3.3. Epicutaneous Immunotherapy (EPIT)

In the case of EPIT, desensitization is achieved transcutaneously by repeated application of the allergen to the skin. The safety profile has been shown to be excellent up to now; no cases of anaphylaxis have been reported. Side effects that may occur with this type of immunization are erythema, pruritus, eczema, and atopic dermatitis at the site of allergen application. EPIT has modest results in terms of inducing food tolerance (28–50%) [62,63,64]. As in the case of SLIT, the main limitation of the method is the amount of allergen that can be administered. Therefore, there is a need to increase the safety profile of the OIT.

### 7.4. Pharmacological Treatment

New pharmacological therapies in the management of food allergies aim at regulating inducing cells and suppressing the native and adaptive responses responsible for triggering allergic reactions [65,66,67]. These therapies are not allergen-dependent, acting nonspecifically, which differentiates them from immunotherapy. The therapeutic arsenal includes targeted biological therapies (Omalizumab, Dupilumab) and microbiome reconstitution agents.

### 7.5. Omalizumab

Omalizumab is an anti-IgE monoclonal antibody. It works by binding to the Fc region of IgE antibodies, blocking the binding to specific receptors on mast cells and basophils [59,60,68], preventing their degranulation and therefore the appearance of allergic manifestations. Omalizumab should be initiated before the OIT and continued for a few more weeks in parallel [31,69,70]. The recommendation considers both the increase of the safety profile of immunotherapy and its effectiveness. Currently, the administration of Omalizumab in the treatment of food allergies is not standardized, so further studies are needed to determine, based on an assessment of the evidence, the duration of treatment. Thus, establishing the optimal time to stop the treatment and the doses that are to be administered, both for immunological pretreatments and concomitant treatments is paramount. Omalizumab can be given to children over six years of age.

### 7.6. Dupilimumab

Dupilumab is a human monoclonal IgG4 antibody that binds to the alpha subunit of IL-4Ra and blocks IL-4 and IL-13 signaling. In 2017, the United States Food and Drug Administration and the European Medicines Agency approved treatment with Dupilimab for adult patients with severe atopic dermatitis whose symptoms are not adequately controlled with topical treatment. Regarding FAs, there are currently two ongoing randomized placebo-controlled Phase II clinical trials evaluating dupilumab [71,72].

## 8. Conclusions

An important goal of caring for patients with FAs should be to maintain quality of life through better knowledge and education of patients and relatives and by moderating the consumption of foods with allergic potential without imposing unnecessary food restrictions. Patients should be instructed to recognize the initial symptoms of an allergic reaction and to be aware of high-risk foods. The possibility of diagnosis by using CRD is important because it can increase diagnostic accuracy, increase the ability to predict the prognosis and the severity of the disease and thereby reduce the specific medication. We need to know in detail the immune mechanisms, diagnosis, and immunotherapeutic options in order to establish strategies for diagnosing, treating, and preventing FAs. The treatment of FAs shows continuous development, especially in terms of biological treatment.

## Figures and Tables

**Table 1:** The most common food allergens, age of onset, cross-reactivity, and range of resolution.

Food	Usual Age at Onset of Allergy	Cross Reactivity	Usual Age at Resolution
Hen’s egg white	0–1 years	Other avian eggs	7 years (75% of cases resolve)
Cow’s milk	0–1 years	Goat’s milk, sheep’s milk, buffalo milk	5 years (76% of cases resolve)
Peanuts	1–2 years	Other legumes, peas, lentils; core activity with tree nuts	Persistent (20% of cases resolve)
Tree nuts	1–2 years; in adults, onset occurs after cross-reactivity to birch pollen	Other tree nuts; co-reactivity with peanuts	Persistent (9% of cases resolve)
Fish	Late childhood and adulthood	Other fish (low cross-reactivity with tuna and swordfish)	Persistent
Shellfish	Adulthood (in 60% of patients with this allergy)	Other shellfish	Persistent
Wheat	6–24 months	Other grains containing gluten (rye, barley)	5 years (80% of cases resolve)
Soybeans	6–24 month	Other legumes	2 years (67% of cases resolve)

Adapted from Nelson Textbook of pediatrics [2].

**Table 2:** Immunopathological classification of FAs.

Immunopathology	Disorder	Clinical Features	Typical Age Group	Prognosis
IgE-mediated	Pollen food allergy	Pruritus, mild edema confined to oral cavity Triggered by ingestion or direct contact	Onset after pollen allergy established (adult > young child) Children > adults	May be persistent and may vary by season Depends on the food
	Syndrome Urticaria/angioedema			
	Rhinoconjunctivitis/asthma	Accompanies food-induced allergic reaction but rarely isolated symptoms It may be triggered by the inhalation of aerosolized food protein	Infant/child > adult,	Depends on food
			except for occupational disease	
		
			
	Gastrointestinal symptoms	Symptoms such as nausea, emesis, abdominal pain, and diarrhea triggered by food ingestion	Any age	Depends on food
		
			
	Anaphylaxis	Rapid progressive, multisystem reaction	Any age	Depends on food
	Food-dependent, exercise-induced anaphylaxis	Food triggers anaphylaxis only if ingestion is followed	Onset in late childhood/adulthood	Presumed persistent
	
Mixed IgE and cell-mediated	Atopic eczema/dermatitis	temporally by exercise Associated with food in 30–40% of children with moderate/severe eczema	Infant > child > adult	Usually resolves
		
			
	Eosinophilic gastrointestinal disorders	Symptoms vary depending on the site of the intestinal tract involved and degree of eosinophilic inflammation	Any age	Likely persistent
		
			
Cell mediated	Dietary protein-induced proctitis/proctocolitis	Mucus-laden, bloody stools in infants	Infancy	Usually resolves
			
	Food protein-induced enterocolitis syndrome	Chronic exposure: emesis, diarrhea, poor growth, lethargy	Infancy	Usually resolves
		
	Celiac disease	Re-exposure after restriction: emesis, diarrhea, hypotension a couple of an hour after ingestion		
	Lactose intolerance			
	Scombroid syndrome			

**Table 3:** The classes of drugs that may influence the result and how long before the test should be stopped.

The Class of Medicines	Time Required from the Last Dose
Histamine H1 receptor antagonists	72 h
Leukotriene receptor antagonists	24 h
β2 stimulant	12 h
Th2 cytokine inhibitors	12 h
Theophylline	48 h
Oral steroids	7–14 days

## Data Availability

Not applicable.
